# Understanding the barriers to identifying carers of people with advanced illness in primary care: triangulating three data sources

**DOI:** 10.1186/1471-2296-15-48

**Published:** 2014-04-02

**Authors:** Emma Carduff, Anne Finucane, Marilyn Kendall, Alison Jarvis, Nadine Harrison, Jane Greenacre, Scott A Murray

**Affiliations:** 1 Primary Palliative Care Research Group, Centre for Population Health Sciences, The University of Edinburgh, Medical School, Teviot Place, Edinburgh, UK; 2 Marie Curie Hospice Edinburgh, Frogston Road West, Edinburgh, UK; 3 NHS Lothian, Waverley Gate, 2-4 Waterloo Place, Edinburgh, UK; 4 Voices of Carers Across Lothian, 8-13 Johnston Terrace, Edinburgh, UK

**Keywords:** End of life, Family carer, Identification, Informal carer, Lay carer, Palliative care, Primary care, Support

## Abstract

**Background:**

Approximately 10% of the UK population have an unpaid caring role for a family member or friend. Many of these carers make a significant contribution to supporting patients at the end of life. Carers can experience poor physical and psychosocial wellbeing, yet they remain largely unsupported by health and social care services. Despite initiatives for general practices to identify carers and their needs, many remain unidentified. Neither are carers self-identifying and requesting support. This study set out to explore the barriers to, and consider strategies for, identifying carers in primary care.

**Methods:**

We integrated findings from three data sources – a review of the caregiving literature; a workshop with researchers who have undertaken research with those caring at the end of life, and focus groups with carers and health professionals.

**Results:**

Three categories of barrier emerged. 1) Taking on the care of another person is often a gradual process, carers did not immediately identify with being a ‘carer’ – preferring to think of themselves in relational terms to the patient e.g. spouse, sibling, son or daughter. Often it was health and social care professionals who encouraged carers to consider themselves as an unpaid carer. 2) As the cared-for person’s condition deteriorated, the caring role often became all-encompassing so that carers were managing competing demands, and felt unable to look after their own needs as well as those of the cared-for person. 3) There was ambiguity about the legitimacy of carer needs and about the role of the primary health care team in supporting carers, from both the perspective of the carers and the health professionals. GPs were thought to be reactive rather than proactive which discouraged carers from asking for help.

**Conclusions:**

The needs of carers have to be legitimised to ensure primary care staff are proactive in their approach and carers are empowered to utilise the support available. Strategies to identify carers have to be sensitive to the complex dynamics of a caring relationship as well as the primary care context. Identification is a key factor in improving support for carers themselves and to enable them to support the patient.

## Background

The Royal College of General Practitioners (RCGP) define a carer as,

“A person of any age, adult or child, who provides unpaid support to a partner, child, relative, or friend who couldn’t manage to live independently or whose health or wellbeing would deteriorate without this help. This could be due to frailty, disability or serious health condition, mental ill health or substance misuse.” [1], pg. 7.

These carers are unpaid and often described as ‘lay’, ‘informal’ or ‘family’ carers [2]. There are approximately 6.5 million carers in the United Kingdom [3], about 10% of the population [2]. Carers can experience poor physical and psychosocial wellbeing and 1 in 5 carers have to give up work as a result of their caring role [2]. According to the 2011 census in England and Wales, carers providing more than 50 hours of care per week, were 3 times more likely to report that their health was ‘not good’ when compared with non-carers [3].

Caring for someone at the end of life can be demanding, both physically and emotionally, for the carer who is also coming to terms with imminent loss of the cared-for person [4]. Support for carers usually needs to be extended to the period after the death of the cared-for person [2], particularly for those ‘at risk’ of poor psychosocial outcomes in bereavement [5]. However, carers are often ambivalent about their own needs and are reluctant to identify themselves as a caregiver in need of support [6]. This may mean they are more vulnerable to poor physical, psychosocial and spiritual wellbeing. Another problem is that the terms carer or caregiver are ascribed to those with a caring role, often by health and social care professionals, and may not be accepted by the carer [7]. Carers may also struggle to continue the caregiving role, which has repercussions for patient and health service provision.

The identification and support of carers is a public health concern. Increasingly, more and frailer people will require complex care [8]. Demand for care from family members for disabled older people will exceed supply by 2017 [9]. The demography of the UK is changing and much of this care will be delivered in the home by family and friends [2], who may be older themselves, and potentially more vulnerable to the demands of the caring role. Policy states that carers should be considered equal partners in care [2]. Supporting carers to care for an individual at home for as long possible – and to die at home, often the preferred place of death [10,11] – has potential savings for the health service. Identifying carers is the first step to supporting them [2], but current strategies are failing to deliver.

Since April 2013, general practices in Scotland are contractually required to have a protocol for identification of carers and a mechanism for the referral of carers for social services assessment, transferred from the Quality and Outcomes Framework (QOF). There is no requirement to identify or refer a particular number of carers. Local enhanced service funding for more detailed carer services in Scotland is no longer provided in most Health Board areas. This means that direct incentives for practices to provide carer services have largely been stopped in Scotland.

The identification of carers is complex – not all family members are carers and not all carers are family members. Patients and carers are not always registered in the same general practice, meaning support for the carers may be overlooked. Therefore the problem is two-fold, general practices do not legitimise carer well-being and are not identifying carers, and carers are not self-identifying in their own surgeries. The aim of this paper is to set out the barriers to, and explore strategies for, identifying carers in primary care and to consider why carers tend not to identify themselves. This paper reflects the findings from the first phase of a feasibility study which was designed to develop a model of identification, assessment and support for carers supporting someone towards the end of life in primary care.

## Methods

We generated, and then triangulated, 3 data sources – (i) A review of the literature; (ii) A workshop with researchers who have undertaken many studies with carers and (iii) Focus groups with carers and professionals. Ethics approval was granted by South East Scotland research ethics committee. Regular meetings were held with the research team (which included GPs, community nurse manager, qualitative researchers and a representative from the local carer organisation) and with an advisory group of current and bereaved carers to discuss emerging themes.

### Literature review

The literature review was designed to answer the following questions:

1. What are the barriers to self-identifying as a carer? (Search terms: family or informal carer and definition; carer and identification; carer and perception)

2. What are the barriers to identifying carers who are providing end of life care in primary care? (Search terms: carer and (end of life or palliative care) and primary care; primary care and identification and carer).

**Figure 1:**
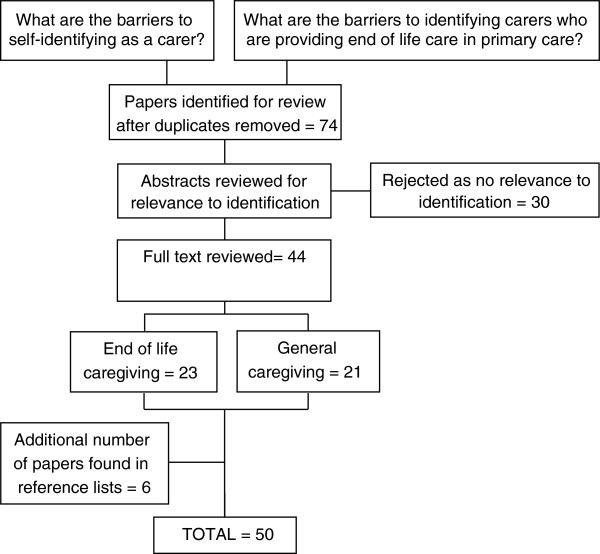
Number of papers included in the literature review.

Searches were conducted in Medline, EMBASE, PsychInfo and ASSIA. Papers from 1998 to May 2013 were included in the review. There were a few key books which were published earlier than this and these were also consulted. Figure 1 shows the number of papers that were included in the review after duplicates had been removed. Quality and relevance were assessed using the following domains: the research focus; caregiver status (bereaved/current); relationship to cared-for person; methods of data collection; population; country of origin; theoretical framework and key findings. After the abstracts had been reviewed for their relevance to the identification of carers, the full texts were read in full and the key themes extracted and synthesised. There were qualitative and quantitative designs, and health and social science papers. Very few papers specifically considered carers’ access to, and use of primary care, although it was sometimes covered in articles about caring more generally. A list of the references used in the review may be obtained from the author.

### Synthesising in-house data

A workshop was held with 7 researchers in the Primary Palliative Care Research Group (PPCRG) who had conducted research with carers, to synthesise their research findings. The discussion focused on the carers’ perception of their role, their identity as a carer and their multi-dimensional support needs (i.e. physical, social, psychological and spiritual) across different illness trajectories. Researchers had undertaken studies about the experience of caring for those with: heart failure, liver failure, chronic obstructive pulmonary disease (COPD), cancer (glioblastoma, lung, colorectal), multiple conditions and those supporting a frail older person [12-19]. The workshop was 2 hours in length and was audio-recorded. Notes were typed up and fed back to researchers to ensure accuracy.

### Focus groups

Five focus groups with carers and health professionals were conducted to develop an in-depth understanding of identification and support-seeking in the primary care context.

### Focus groups with carers

Focus groups were conducted with spousal carers (n = 4), bereaved carers (n = 5) and offspring carers who were supporting a parent (n = 6). Table 1 shows the characteristics of the carers. Participants were identified by a local carer third sector organisation; from the local hospice; and through specialist nurses at the local teaching hospital. Potential participants were given an invitation letter and information sheet and their details were passed to the project researcher (EC), who followed up with a telephone call. Written consent, including permission to record the focus group, was gained immediately before it commenced. A broad topic guide was used.

**Table 1:** Focus group participants – carer characteristics

**Relationship to cared-for person**	**Illness of cared-for person**	**Current or bereaved carer**
Wife	Vascular disease	Bereaved
Wife	Cancer	Bereaved
Daughter	Alzheimer’s disease	Bereaved
Wife	Stroke	Bereaved
Husband	Stroke	Bereaved
Husband	Multiple sclerosis	Current
Wife	Multiple sclerosis	Current
Husband	Dementia	Current
Wife	COPD	Current
Daughter	Dementia	Current
Daughter	Dementia	Current
Daughter	Dementia	Current
Daughter	Cancer	Current
Daughter	Frailty	Current
Daughter	Alzheimer’s disease	Current

The focus groups with spouses and offspring carers explored barriers to being identified as a carer; the point at which (if any) carers begin to see themselves as a carer; what support was available to them and their perceptions of the support required to protect their health and well-being. The focus group with bereaved carers explored when in the caring trajectory they felt like a carer, the support they experienced during the cared-for person’s illness and whether (and at what point) they felt additional support might have been helpful.

### Focus groups with professionals

Two focus groups were conducted with health professionals (n = 8). Table 2 shows the numbers and role of the professionals. The focus groups included GPs, a specialist carer co-ordinator for the local Community Health Partnership, community specialist palliative care nurses and district nurses who had a particular interest in palliative care. Written consent was gained before the focus group commenced. The groups explored current practices in carer identification and support and how links with local carer organisations can be best utilised.

**Table 2:** Focus group participants - number of health professional participants

**Health professional**	**Number**
District nurse	2
General practitioner	2
Carer co-ordinator for community health partnership	1
Community specialist palliative care nurses	3

The focus group discussions were fully transcribed and analysed in QSR Nvivo 9 [20], using content analysis [21]. Specific focus was given to the barriers to identification, assessment and support, and to the organisation of services. Analysis of the carer focus groups also considered the caregiving journey, which allowed us to reflect on transition points e.g. when the carer felt they took on the caregiving role. We adopted a constructionist perspective to analysis, where attention was paid to areas of agreement and disagreement within the groups [22]. This technique highlighted the broad spectrum of caregiving experience. The data were anonymised and pseudonyms were applied.

### Triangulating the data

The findings from the 3 sources were compared, contrasted and corroborated to improve the ‘rigour, breadth, complexity, richness and depth’ of the inquiry [23] pg. 5.

## Results

Three factors emerged as barriers to carer identification:

1) The gradual process into caring, and the transition to, and identification with, the term ‘carer’.

2) The all-encompassing nature of caring for someone with advanced illness often resulted in carers prioritising the needs of the ill person at the expense of their own.

3) The legitimacy of carer needs and ambiguity of the role of primary care teams in meeting them.

### Caring as a gradual process

#### Identifying with the term ‘carer’?

The 3 sources of data highlighted that those with a caring role did not necessarily identify with the term ‘carer’, preferring to think of themselves as relatives or friends who were sharing the journey with the ill person. Researchers at the workshop reported that they sometimes avoided using the term ‘carer’ when recruiting carers to research studies.

The literature revealed that the transition into caring was often a gradual one and was associated with a wide range of responsibilities [24,25]. Small duties accumulated until carers recognised that they were undertaking a significant supportive role – particularly if they had to give up work at that point [26]. Many carers saw the caring role as intrinsic to their relationship as wife, husband, son, daughter, sibling etc. [26-32]. Over time, some carers did identify with the term carer, yet others rejected it completely [26]. Some caregivers preferred to be self-sufficient, feeling that they did not yet need help [24], or that support from external paid carers was a potential intrusion [27,33]. Furthermore, acknowledging the caregiving role meant recognising that the ill person needed to be cared for [26]. In some circumstances neither the carer, nor the cared-for person may be ready to face this reality.

The focus group participants discussed when they felt they became carers. Faye felt she became her mother’s carer when her mother lost confidence in her activities of daily living, and subsequently her independence. Faye described the reversal of roles that took place as the caring role evolved.

“Where I feel the carer became what was the daughter, the carer part became more relevant, was when she lost that confidence.” (Faye, caring for her mother).

For some, it was only when the caring role was acknowledged by others that they realised they were ‘carers’ [34]. For example, Alison’s GP told her that she was a carer and therefore could be on the carer register.

“Alison: it was the GP, my GP, who said “you are a carer and I need to put you on the register” and this…

Researcher: Ah, interesting, OK.

Alison: And I was going “oh!” And so it was news to me, you know, I just assumed I was the daughter” (Alison, cared for her father).

The focus group participants highlighted that acknowledgement and recognition of their caring role often occurred when there was a crisis, e.g. if the illness of the cared-for person deteriorated markedly, and the extent of their caregiving role came to light. However, recognition could be positive. For example, being identified as a carer meant Alison was entitled to particular benefits through her employer. Workshop researchers reflected that following a crisis, it was sometimes a relief for carers to be offered a package of paid care e.g. when the cared-for person returned home from hospital.

However, for others, being termed a carer could be seen as detrimental or offensive.

“She [carer] loved him [patient] dearly, they loved each other dearly, and she’d been providing *such* an amount of care. She was getting him up, helping him with the shower, you know, he couldn’t go to the toilet without her, she was there all the time, she was in a carer role. And when I met them and I was asking about benefits and I said something and remarked that she was a carer. And she sat up really straight and she said, and she got really flustered and said “oh, am I a carer now?” Because he’d been living with these strokes and she’d been doing this, you know, the disability from the strokes for so long, suddenly he’s got lung cancer and he gets referred to us [specialist palliative care]. And I said “carer” and she was really shocked by that! And that made me think “oh, crumbs!” you know, the word ‘carer’ has such a big weight” (Health professional, community specialist palliative care nurse).

The caring situation is complicated and what is right for one family may not be right for another. Some carers may welcome paid care and others may find it an intrusion, making a private place, public. If carers do not recognise themselves as such, they are less likely to seek out support which suits their needs.

#### Changing relationships in the context of caring

How existing relationships could be challenged by the caring situation was highlighted in all 3 data sources – particularly by focus group participants who were caring for a parent. For example, Pamela was the main carer for her mother who suffered from dementia. Pamela’s mother did not accept her need for care and still wanted to be independent, so it was difficult for Pamela to ask for help and explore the services available to her and her mother. This increased Pamela’s feelings of isolation and concern. Pamela also highlighted the challenge of managing existing relationships with family and friends. Pamela’s feelings were echoed by other dementia carers in the study.

“But I do feel that relationships slide, both with your children, with your husband or your partner, I just think it all just… And I think that, you know, your children and your partner or your husband maybe understand initially but when it’s for some years, you know, it begins to bite.” (Pamela, caring for her mother).

Relationships were further compromised if carers were experiencing stress, anxiety and tiredness. Researchers at the workshop highlighted that the relationship between the patient and the carer may be challenged if the carer thinks they are caring, but the patient does not think they are being cared for. This could be another reason why carers remain unidentified and potentially unsupported. Joan described her frustration as her husband was a perfectionist by nature, but she saw that compromises had to be made in light of his deteriorating condition.

“Like yesterday morning, I sort of had like been sort of, getting annoyed about things because he’s a perfectionist and I said things just can’t be perfect now” (Joan, caring for her husband).

The relationship between the carer and the cared-for person could affect the carer’s willingness to present themselves as someone in need of support – particularly if the cared-for person is resistant to, or does not think that help is needed [24].

### Engulfed by the caring role

Findings of the literature review and focus groups suggest that caring, particularly towards the end of life, can be all encompassing [24,27,35,36]. Carers may be engulfed by the process and unable to access support because they do not have sufficient time, or are fearful of leaving the cared-for person [33,37]. Carers may also be concerned about diverting resources to themselves at the expense of the cared-for person [38]. As the patient’s illness progresses the carer may struggle to find the time to access support for themselves or visit the GP with their own medical concerns.

Carers in the focus groups discussed how their social world often contracted, particularly as they became fearful of leaving the cared-for person,

“My husband would say to me “oh yes, you must go into town and meet [friend]” – my friend who… I was just a bundle of nerves, if the bus was held up coming back” (Joyce, cared for her husband).

Many carers in the focus groups commented on how isolating the caring role was,

“I feel very isolated, there is only me. She [mother] won’t have any kind of help of any description and my siblings don’t really want to know. So very much on my own” (Pamela, caring for her mother).

This feeling of isolation compromised the carers’ own identity and sense of self.

“I’d always had fairly wide interests, I found that I was losing interest in anything in sport or politics or whatever.” (Michael, caring for his wife).

“You do have to really work at maintaining your own sense of self, you know, and that you have a value outside of your role towards this other person” (Jane, cared for her husband).

#### Managing competing demands

The focus group participants highlighted that the all-encompassing nature of caregiving at the end of life also complicates the delicate balance of competing demands that the carers experience in all four domains; physical, psychological, social and spiritual. They highlighted the challenges of managing their caring role and friends, family and work. Researchers described how it became more of an issue as the cared-for person’s condition deteriorated.

Alison struggled with the demands of work, family and the caring role for her father, yet at the same time she wanted to spend more time with him before his death.

“And so it made me… you know, when you were thinking, maybe I shouldn’t be at work, maybe I should leave, and the GP – his GP – you know, was saying “oh, you must keep your work, you can’t…” you know, and so they were stressing it and I’d think well, that’s fair enough but how do I do this as well?” (Alison, cared for her father).

Joan described how she gave up her demanding job as the caring role gradually increased. As a result, she had no time to herself and decided to do voluntary work to create distance between herself and the caring situation.

“I’d gave my work up to look after my husband and I felt I wasn’t doing anything for myself” (Joan, caring for her husband).

Researchers at the workshop commented that offspring who were caring for a parent found managing demands on them more challenging. However, a spouse in the focus group described how she did not want to burden her children, who themselves had young families with her concerns about the caring role.

The all-encompassing nature of caregiving is a potential barrier to identification. There is evidence in this section to suggest that as carers struggle with demands on their time, they may be unable to recognise their own needs as legitimate and seek support.

### The role of primary care in legitimising need

All 3 data sources suggested that primary care professionals’ lack of knowledge about existing services for carers was a barrier to providing support. Also, in the literature review and in the focus groups, GPs were thought to be reactive, rather than proactive in their approach to supporting carers [39,40]. Moreover carers did not know what, if anything, to look for and were dependant on a thoughtful and knowledgeable response from professionals [41]. Professionals were not seen to legitimise carer needs. One carer described how she thought there was nothing for her, and another, that not knowing where to go for help was the problem,

“I just don’t feel there’s… for me, I don’t feel there’s *anything*” (Pamela, caring for her mother).

“And it’s not knowing where to look for help that is the problem” (Jane, cared for her husband).

#### Role ambiguity

The availability and provision of services was complicated by ambiguity about the role of primary care professionals in identifying and supporting carers, both from the point of view of the GP and the carer [39,42]. The GPs and district nurses in the focus groups thought that identifying and supporting carers was their role, however the carer themselves may not.

“Well, certainly as a GP, I would certainly see it as a very important part of my role to help someone who is a carer with that issue, but I think *they* would struggle to see it as the GP’s role” (Health professional, GP).

Researchers described the mixed views that carers had about support from external care. For example, some of those caring for someone with COPD found that external carers could be intrusive and impersonal. If patients or carers are reluctant to have external care provision in the home they may not identify, preferring instead to be self-sufficient. Although, health professionals in the focus groups acknowledged a responsibility to identify and support carers, the literature suggests that the challenges of doing so include issues of confidentiality, differing information needs between patients and carers and when the carer is registered with a different general practice [42]. It was also noted that health professionals tend to see the carer in relation to the patient [31,39], meaning the individual needs of the carer may be overlooked. One GP in the focus group thought that as caring was not, in itself a medical problem, the carer may not see their needs as legitimate for the GP and therefore not self-identify.

“But I’m aware that, the same with people when they come to the GP, they think they have to do it with some kind of medical problem, or they at least have to be officially depressed or something” (Health professional, GP).

Limited resources were cited as a common barrier to identifying and supporting carers in the literature, where practitioners were concerned about opening ‘Pandora’s box’ and being overwhelmed by demand for services that may not be available [43], or that they lacked time [39]. This was echoed by professionals in the focus groups. In contrast, carers’ perceived needs are often relatively modest [43]; rather carers often want acknowledgement of their role, to be listened to and to gain access to other sources of support [35,44], as opposed to major service provision.

#### Communication

Workshop discussions highlighted how researchers were often confronted with a complex network of people involved in a caring situation, making it difficult to establish whom, if anyone had the main caring role. This may also apply in the general practice context, although such a network of carers might support each other and therefore have less need for formal support.

Most professionals acknowledged that finding time to speak with the carer was difficult, and those who worked in the community highlighted the importance of the door-step conversation.

“You kind of can put paranoia to the patient then because they feel ‘what are they talking about?’ and…And I’ve had that with patients before is, you know “what were you talking about? You know how long I’ve got left” and “you know more than you’re letting on” and stuff.” (Health professional, community specialist palliative care nurse).

Ambiguity about whose role it is to identify carers in the community, from the perspective of carers themselves and professionals is a barrier to identification. This is complicated by lack of time, resources and knowledge of available services.

## Discussion

The number of carers required to support an ageing population will continue to increase. These carers will require physical, practical, and psychosocial support, but how can health professionals help them when they remain unidentified? Promoting carer identification is a vital first step, as only then can carers who might benefit from support; a) be acknowledged for the role they undertake, b) have their needs sensitively assessed and c) receive individualised support. This paper adds to the existing body of knowledge as it addresses the barriers to identifying carers from 3 sources of data.

The challenge of identifying the carers of those with advanced illness in primary care is two-fold; barriers that stem from the carers themselves, and barriers that stem from primary care. The needs of carers necessitate legitimisation for both professionals and carers. Given this complexity, a two-prong strategy to target carers and general practices is needed. Raising the profile of available support for carers should encourage carers to self-identify and request help. At the same time, practices need be proactive in looking for carers within their practice population. Patients and their carers who are registered in different general practices presents an obvious challenge [42], but can be addressed by empowering carers to self-identify.

The carer’s perception of their role and where they are on the caring trajectory may influence their willingness to identify. Carers sometimes felt they did not need help at the time they were asked, yet a later crisis in the caring situation may have been preventable if support systems had been put in place earlier. This was also described by Adams (2006) in the context of the caregiving trajectory in dementia [24]. Although health professionals cannot force support on carers, they can encourage them to seek advice and support when their situation changes.

The caring role is complex, and this paper has illustrated a number of reasons why carers may not self-identify. For example, carers may prioritise the needs of the cared-for person [45], experience feelings of guilt, and question the legitimacy of their needs [46]. Carers may feel that their home will become medicalised and no longer feel like their own if they have equipment or external carers to help them. They may feel best able to meet the needs of the patient and that the patient does not want help from outside the family. Accepting outside help may mean facing up to the fact that the patient is seriously ill [26]. Therefore, working with, and recognising carers as equal partners early in the illness trajectory may help to alleviate some of these issues [47]. Anticipatory care planning involving carers could encourage them to think about the future and put strategies in place to manage competing demands. Community nurses may have a particularly good relationship with carers and are in a unique position to identify carers, regularly assess their needs and monitor their overall health and wellbeing. More generally, health and social care professionals need to engage with the network of carers if there is one. Medical records should keep data on the extent of the care provided by the caring network using a relationship circle or family tree [48].

### Education

Professionals often lack knowledge about the wide range of services available to carers. The presence of a carer expert in general practices could address this knowledge deficit. RCGP have already recognised the gap in this area and have appointed nine carer champions in England who provide expert advice to carers. Many areas have a range of different carer organisations that offer information, signpost to support as well as providing direct support. A range of mechanisms for identification are recommended; for example pharmacists could play a role, as could other health and local authority providers. Practices should also ensure that all new patients are asked at registration if they are, or have a carer, or a caring role. They can then be coded accordingly and added to a register of carers so that the needs of the carer can be considered during any consultation.

If primary care teams advertise the services available to carers, more carers are likely to identify themselves as such. Suitable places for carer information include: practice websites; posters in the waiting room, in the pharmacy or with the district nursing team who regularly visit the home. Although these may be in use already, their efficacy is yet to be determined. Community teams need to work together to achieve this and to be well informed of local services. Hirst (2002) commented that professionals are able to best meet the needs of carers when they provide high quality advice and information [49]. This will include signposting to other agencies such as local carer services and specific sources of community support, such as social services and benefits advice. Primary care need to develop direct links with social care agencies to ensure they have up-to-date knowledge of the services available to carers.

### Limitations

This paper reports the first, theory-building phase to assess the feasibility of a complex intervention to identify, assess and support carers in primary care. One of the main strengths of this article is the triangulation of 3 data sources. However, the specific context of each of these data sources means that the findings may not be applicable in other settings (e.g. different ethnic groups). The literature review explored relevant theory and provided evidence that the intervention which has been modelled is warranted. The researcher workshop demonstrated extensive experience in conducting longitudinal and multi-perspective research with patients and their carers.

The focus groups generated data from current and bereaved carers and professionals to ensure a recent view of the carer experience. Eleven (73%) carer participants for the focus groups were recruited through a local carer organisation and their experiences may have differed from that of carers not in contact with carer agencies. All the carers were/or had been a blood relation to the cared-for person therefore friends or neighbours with a caring role were under-represented. All carers were supporting a family member with a life-limiting illness but not all the patients were at the end of life. For this reason we conducted 1 focus group with bereaved carers. The health professionals who participated in the focus group may have self-selected as they had particular experiences of, or ideas for, carer support meaning the sample was potentially biased.

## Conclusions

This paper explored the barriers to, and suggested strategies for, identifying carers in primary care using 3 sources of data. The findings suggest that identifying carers needs a two-prong approach, where primary care proactively identify carers, and at the same time, empower carers who cannot be identified through the cared-for person to self-identify. The findings also suggest that early intervention is needed to prevent carers experiencing crisis, and poor physical and psychosocial health. Identifying carers is the first step to supporting them directly, and supporting them to continue caring. However, success is dependent on the legitimisation of carer needs to primary care, other health and social care professionals, and to the carers themselves.

## Competing interests

The authors declare that they have no competing interests.

## Authors’ contributions

All authors made substantial contributions to conception and design and have been involved in drafting the manuscript or revising it critically for important intellectual content. EC conducted the literature review, convened and facilitated the researcher workshop, conducted the focus groups and analysed the data. EC drafted the manuscript. AF, MK, AJ, NH, JG and SAM made substantial contributions to conception and design of the study. JG was involved in recruiting participants for the focus groups. All authors contributed to the interpretation of data. All authors read and approved the final manuscript.

## Pre-publication history

The pre-publication history for this paper can be accessed here:

http://www.biomedcentral.com/1471-2296/15/48/prepub
